# Discontinuation of brace treatment in adolescent idiopathic scoliosis (AIS): a scoping review

**DOI:** 10.1007/s43390-024-00882-3

**Published:** 2024-05-01

**Authors:** Lizzie Swaby, Mengwei Cui, Ashley Cole

**Affiliations:** 1 ScHARR, University of Sheffield, Sheffield, S1 4DA UK; 2 Department of Paediatric Orthopaedics, Sheffield Children’s Hospital, Sheffield, UK

**Keywords:** Adolescent idiopathic scoliosis, Brace discontinuation, Brace weaning, Scoping review

## Abstract

**Purpose:**

Brace treatment for adolescent idiopathic scoliosis (AIS) is usually prescribed for 20–40° curves in patients with growth potential. The aim is to reduce the risk of curve progression during growth and to avoid the curve reaching a surgical threshold. Having as small a curve as possible at skeletal maturity will reduce the risk of curve progression during adult life. While evidence exists for brace treatment in AIS, there is disagreement on how and when to discontinue bracing. The purpose of this review was to investigate what criteria have been reported for initiating brace cessation and published weaning protocols and to look at estimates of the number of patients that may progress > 5 degrees after the end of growth.

**Methods:**

This scoping review summarizes existing knowledge on the best time to stop bracing in AIS patients, how to “wean,” and what happens to spinal curves after bracing. Searches were carried out through MEDLINE, EMBASE, and PsycINFO in April 2022. A total of 1936 articles were reduced to 43 by 3 reviewers. Full papers were obtained, and data were extracted.

**Results:**

Weaning was most commonly determined by Risser 4 (girls) and 5 (boys). Other requirements included 2 years post-menarche and no growth in standing/sitting height for 6 months. Skeletal maturity assessed from hand and wrist radiographs, e.g., Sanders’ stage; distal radius and ulnar physes, could determine the optimal weaning time to minimize curve progression. Complete discontinuation was the most common option at skeletal maturity; variations on weaning protocols involved gradual reduction of bracing over 6–12 months. Curve progression after weaning is common. The 12 studies reporting early curve progression after brace weaning found a mean Cobb angle progression of 3.8° (*n* = 1655). From the seven studies reporting early curve progression by > 5 degrees, there were 236/700 (34%) patients. There is limited information on risk factors to predict early curve progression after finishing brace treatment with larger curves, especially those over 40 degrees possibly having more chance of progression.

**Conclusion:**

Curve progression after bracing cessation is a negative outcome for patients who have tolerated bracing for several years, especially if surgery is required. The literature shows that when to start brace cessation and weaning protocols vary. Approximately 34% of patients progressed by more than 5 degrees at 2–4 years after brace cessation or weaning. Larger curves seem more likely to progress. More research is needed to evaluate the risk factors for curve progression after brace treatment, defining the best time to stop bracing based on the lowest risk of curve progression and whether there is any benefit to weaning.

**Supplementary Information:**

The online version contains supplementary material available at 10.1007/s43390-024-00882-3.

## Background

### Adolescent idiopathic scoliosis

Growth is the major factor for worsening of scoliosis. When a patient has finished growing, if their curve is less than 50 degrees, curve progression is less likely and rarely causes problems. Curves over 50 degrees after growth have a high chance of further progression, which can lead to cardiorespiratory morbidity and pain into adulthood [2]. Curves this large are usually treated surgically. Surgery is often successful, but carries risks to the patient and high cost to health-care providers.

### Brace treatment

There are two main options for reducing the risk of curve progression during growth; scoliosis-specific exercises, where there is limited evidence, and, brace treatment, where a rigid, plastic brace is worn around the torso. While different manufacturers produce these braces to a slightly different design, all braces primarily seek to reduce the curve size in-brace to decrease the chance of progression, until the point the patient has stopped growing [3, 4]. The efficacy of bracing is well established, although acceptability, and therefore compliance, can be an issue in this patient population [5, 6]. In some cases, patients may still require surgery to correct the curve, either if the curve progresses during growth or if the curve progresses once bracing has been discontinued.

### Purpose of this review

As curve progression is linked to patient growth, it is vital to maintain bracing until skeletal maturity, or the end of growth, is reached. While it may be desirable to brace patients for as little time as possible due to adverse effects on mental health and quality of life [7], early removal of the brace when there is still growth potential can result in curve progression. There are a number of criteria that can be used to determine the point at which skeletal maturity is reached, with no universal agreement on the most appropriate. In addition, there is no clinical consensus on how to stop bracing. Roye et al. (2020) reported the results of a Delphi study to develop best practice guidelines for the use of bracing in AIS. Regarding weaning, they concluded that there was low-level evidence (Grade C) to recommend using Sanders stage, Risser stage, change in height, curve magnitude, and curve progression when considering discontinuing bracing [8]. Also, once the decision to stop bracing has been made, there should be a weaning period of at least 6 months before fully discontinuing the brace.

This scoping review aimed to summarize the existing literature on when to cease brace treatment, how to carry out this discontinuation of treatment, and what happens to spine curves after the end of bracing.

## Methods

A scoping review methodology was chosen for this review to keep the data extraction broad, and to be able to provide a summary of the existing literature in relation to the objectives, and identify knowledge gaps where further research may be needed [9]. Different patient populations, skeletal maturity criteria, weaning protocols, and follow-up would make a formal systematic review or meta-analysis misleading.

This review has been reported in line with PRISMA-ScR [10].

Articles were eligible for inclusion in this review if they reported primary data collection; for patients diagnosed with AIS, including information on the participating subjects at the start of bracing (e.g., age, skeletal maturity, curve size). Studies needed to provide information on one or more of the following:

- When to stop brace treatment
- How to stop or wean from brace treatment
- Reporting on curve progression up to at least 1 year after brace cessation.

Studies reporting on infantile or juvenile idiopathic scoliosis were not included, unless they reported outcomes separately for AIS patients. Similarly, articles reporting on patients with other comorbidities were excluded as there may be other causes for the scoliosis. Review articles, protocols, and conference articles were not included. There was no restriction on study design, year of publication, or country; however, some articles could not be obtained in English translations at full text and could not be included.

Literature searches were conducted through Ovid (MEDLINE, EMBASE, and PsycINFO) from database inception to April 2022. Where articles were needed for full-text review, but were not freely available, these were requested through institutional library services and contact directly with the authors. The full search strategy is available in Appendix 1.

Screening was carried out independently, in parallel by two reviewers (LS and MC), with the third reviewer (AC) resolving any disagreements. Once title and abstract screening and then full-text screening were complete, data extraction was completed. This was completed in full by one reviewer (MC) with 20% checked for agreement by a second reviewer (LS). There were no disagreements, so no further data extraction was completed by reviewer two. Most papers did not record whether the end-of-treatment radiograph was taken in- or out-of-brace. None of the papers had inconsistent findings from pre-bracing to end-of-treatment to follow-up that might suggest that any reports were of in-brace radiographs at the end of treatment.

Risk-of-bias assessment was not completed for this review [10].

Results were synthesized and presented in narrative format. The full data extraction tables are available in Appendix 2.

Gap analysis was performed systematically looking at the recommendations from the included articles.

## Results

### Study selection

**Fig. 1:**
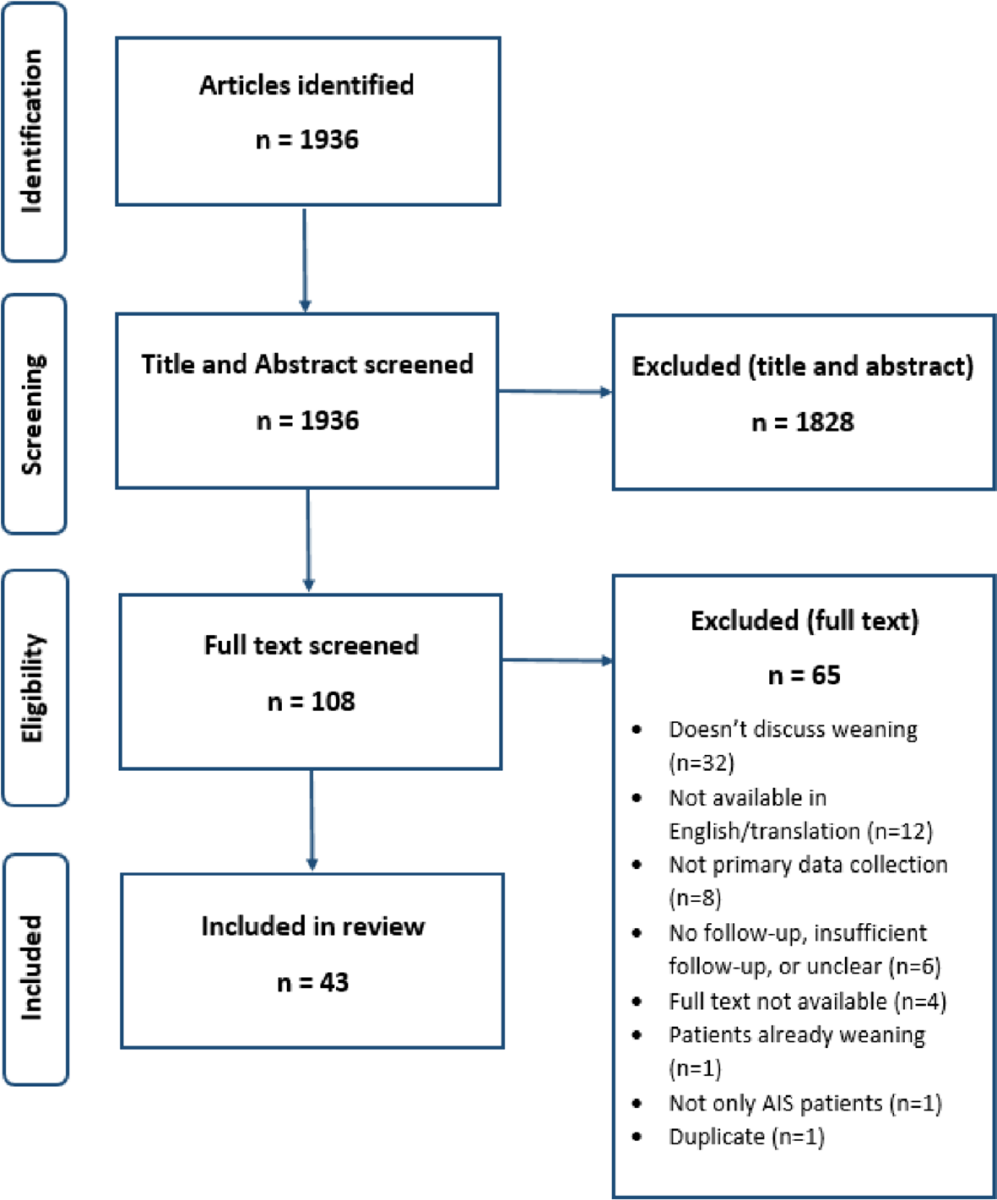
PRISMA-ScR flowchart

Of the 1936 articles identified, 1828 were excluded on title and abstract review, leaving 108 reviewed at full text. A further 65 were excluded, and 43 articles contributed to the data presented in this review. Full details can be found in Fig. 1.

Where articles were not available in full text, contact was made with the authors to obtain a copy. Where responses were not received, articles were considered as not available for this review. Similarly, efforts were made to locate copies or translations of papers not written in English, but there were some of these that could not be obtained.

### Study characteristics

Most of the included articles were published between 2011 and 2020 (*n* = 19), with the earliest in 1986 and the latest in 2022. More included studies were published by teams in Italy (*n* = 8), followed by China (*n* = 7) and the USA (*n* = 7). Three studies were conducted in each of Denmark, Norway, and Sweden, with two in each of Canada, Greece, and Finland. Other studies were published in the Netherlands, Japan, Poland, Israel, Spain, and Lebanon (each with *n* = 1).

There was an almost even split between retrospective (*n* = 19) and prospective (*n* = 24) data collection methods; 22 studies included more than 100 participants each and 21 included less than 100 participants in their studies. Ten studies included only female participants.

The studies included had the following study aims:

- Effectiveness of a particular brace in the treatment of patients with AIS (*n* = 16);
- Predictors of curve progression during brace treatment (*n* = 9);
- Effect of bracing strategies on long-term spine curvature (*n* = 6);
- Criteria for skeletal maturity and timing of brace cessation (*n* = 3)
- Effect of brace compliance on curve progression (*n* = 2);
- Miscellaneous (*n* = 7).

### When to discontinue brace treatment?

The end-of-brace treatment is usually determined by skeletal maturity, and in the 43 articles included in this review, criteria for defining skeletal maturity were varied. Most commonly, this was through assessment of Risser stage, with Risser 4 or above considered skeletally mature for girls and Risser 5 for boys; change in sitting or standing height or arm span of less than 1 cm over a 6-month period; 18 or 24 months post-menarche for female patients; or Tanner classification of 4 or 5. Other studies used different thresholds including completion of fusion of the ring-apophysis on the lateral spinal X-ray; 24 to 30 months after menarche; Risser grade 3; female patients aged 16 and male patients aged 18; skeletal hand age of 14 years for female patients and 16 years for male patients (i.e., Sanders’ 8); or no worsening of the scoliosis during the 24-month follow-up. Sanders’ stage 7 or 7b (Sanders’ 7 plus ulnar grade 8) or combinations of distal radius and ulnar grades (radius grade 9 and ulnar grade 8) are suggested to reduce the risk of curve progression after weaning. Some articles used a combination of the above criteria, two articles did not state the criteria used for discontinuation of bracing, and two articles stated that brace cessation commenced at skeletal maturity but did not define skeletal maturity.

### How to discontinue brace treatment?

Sometimes bracing is ceased when the selected threshold is reached, and in other cases, the brace treatment may be stopped gradually through a period of weaning. Of the articles included in this review, 30 discontinued brace treatment through weaning and 11 stopped bracing altogether straight away. Two articles did not specify how brace treatment was discontinued.

Weaning protocols had some similarities, but were varied. Three articles by one author reduced brace wear time by 2 to 4 h every 4 months [11–13]; another article reduced wear time by the same amount but at 2-month intervals [14]; and three studies used progressive weaning over 6 months, although only one described this process in detail [15]. Other authors described a reduction in brace wear to 16 h per day and then 8 h per day but did not specify over what time period [16]. Two studies reported reducing wear time to 12 h per day over 1 year, followed by night-time-only wear for 1–2 years [17, 18].

Other articles only discussed the weaning time, and not how this was done; weaning time was 4 weeks [19] or 1 year [20]. Another article reported a relatively short weaning process reducing brace wear by 2 h every week for 2–3 months [21]; while Shi et al. and Wiley at el reduced brace wear to night time only for between 6 and 12 months [22, 23]. Some articles did not use time as a determinant of the weaning process, but rather weaned gradually until Risser stage 5 was reached [24].

Three studies included patient exercises alongside brace weaning [16, 24, 25].

### What happens to the spine curve after brace treatment is stopped?

SRS criteria published in 2005 [4] specify a need to follow up patients for 2 years after brace treatment. Curve progression after skeletal maturity (end-of-treatment, end-of-bracing) can be recorded as:

- The mean number of degrees of progression;
- The number (percent) of patients progressing more than 5 degrees, with many studies considering a change of more than 5 degrees a treatment failure.

There were 28 studies reporting the difference in curves in degrees, 7 of which reported a mean curve progression greater than 5 degrees and 19 with a mean change of less than 5 degrees; two studies reported on subgroups which had mean curve progression equal to 5 degrees.

Curve progression ranged from 0 to 14.1 degrees in the 28 articles reporting mean change in degrees after brace weaning, but follow-up ranged from 1 to 25 years, making it difficult to distinguish early and late curve progression. There were 12 studies evaluating the mean curve progression 1–3 years after the end-of-full-time brace treatment (one study evaluating night-time bracing was removed). Table 1 summarizes these 12 studies which reported a mean progression of 3.8 degrees (range 0—8.3 degrees) [12, 22, 25–34].

**Table 1:** Studies reporting mean curve progression 1–3 years after stopping brace treatment in AIS patients (full-time rigid braces only)

Paper	Bracing inclusion	n	Cobb angle at start of bracing	Cobb angle at weaning/brace stop	Cobb angle at FU	Progression after weaning/brace stop	Weaning indications	Weaning method	Follow-up	Comments
Appelgren & Willner 1990	Not stated	121	32.0 ± 8.0	22.0 + -/9.0	28.0 ± 10.0 (1 year) 30.0 ± 10.0 (2 years)	6.0 ± ? (1 year) 8.0 ± ? (2 years)	Not stated	Not stated	1 year + 2 years	
Montgomery 1990	Not stated	168	33.2 ± 6.5	28.0 ± 10.7	32.5 + -/10.5 (2 years)	4.5 ± ? (2 years)	Not stated	Not stated	2yr	End of bracing described as end of weaning = patient out of brace in the day 0.6 ^o^ progression from 2 to 6.9yrs after weaning
Upadhyay 1995	CA 20–45 Risser 0–3	52	33.0 ± 7.0	23.0 ± 10.2	28.0 ± 8.2	5.0 ± 6.1	Risser 4	Reduce to 18h for 1 month, 14-16h for 2 months, night-time for 2–3 months	2 years + (mean 3 years)	
Yrjonen 2007	CA > 25	51m 51f	33.1 32.4	31.4 27.0	34.8 32.0	3.4 5.0	Risser 4 growth ceased	Not stated	Mean 2.4 years	
Zaina et al. 2009	Not stated	68	26.8	21.8	23.1	1.3 ± ?	Risser 3	Gradual reduction 2–3 h/day until night only at 6 months	2.7 years	Exercises during weaning
Aulisa et al. 2012	CA25-40 Risser 0–2	40	26.4 ± 2.8	8.4 ± 5.2	11.6 ± 7.7	3.2 ± 2.0	Ring apophysis fusion on lateral radiograph	2–4 h reduction every 4 months	1 year	Lumbar only––SD calculated from 95% CI 2.2° curve progression between 1 and 4.5 years
Brox et al. 2012—Compliers	CA > 20	355	33.1 ± 7.2	26.4 ± 9.5	27.4 ± 9.2 (1 year) 28.1 ± 9.2 (2 years)	1.0 ± ? (1 year) 1.7 ± ? (2 years)	2-year post-men or Risser 4 or 5	Not stated	1 year + 2 years	Self-reported > 20 h/day
Brox 2012 – non-compliers	CA > 20	82	32.8 ± 7.6	33.5 ± 10.2	33.7 ± 10.4 (1 year) 33.2 ± 9.9 (2 years)	0.2 ± ? (1 year) -0.3 ± ? (2 years)	2-year post-men or Risser 4 or 5	Not stated	1 year/2 years	Self-reported < 20 h/day
Aulisa et al. 2015	CA 25–40 Risser 0–2 < 1 year post-men	102	31.5 ± 4.3	16.6 ± 9.0	16.3 ± 9.6	-0.3 ± 2.6	Ring apophysis fusion on lateral radiograph	2–4 h reduction every 2 months for 8–10 months	1.1 years	SD calculated from 95% confidence limits
Shi et al. 2016	CA 20–40 Risser 0–2 < 1 year post-men	200	27.7 ± 5.9	30.1 ± 10.4	33.6 ± 10.7 (1 year) 35.0 ± 11.2 (2 years)	3.5 ± 5.8 (1 year) 5.1 ± 6.5 (2 years)	Risser stage 4 and more than 2 years post-men and no growth between two visits	Night wearing for 6 months	1 year + 2 years	0.6 ^o^ progression from 2 to 4.3 years after weaning
Cheung et al. 2019	CA 25–40 Risser 0–2 < 1 year post-men	144	32 ± 5	35.5 ± 7.3	Not reported	8.3 ± 3.0	Risser Stage 4, no growth in stand/sit height/arm span in 6 months, at least 2 years post-men	Stop + discarded	2 years + (mean 3 years)	All stopped using brace at least 24 h before X-ray
Grothaus et al. 2020	CA 20–40 Risser 0–2	42	-	-	-	6.0 ± 5.0	Risser 4; no height change	Stop	2 years	Progression independent of Sanders 7 before or after brace stopping
Cheung & Cheung 2021	CA 25–40 Risser 0–2 < 1 year post-men	179	-	34.6 ± 7.7	37.9 ± 9.8	3.3 ± ?	Risser Stage 4, no growth in stand/sit height/arm span in 6 months, at least 2 years post-men	Not stated but usual in the unit to stop and discard	2 years	Different cohort from 2019 study All stopped using brace at least 24 h before X-ray 0.6 ^o^ progression from 2 to 3.4 years after weaning
Summary		1655				Mean Cobb angle progression 3.8° *				

*CA *Cobb angle

*Men* menarche

? unknown standard deviation

^*^2-year follow-up when 1- and 2-year follow-up given

Four studies continued follow-up beyond 1 or 2 years up to 6.9 years (Table 1), showing 0.6–2.2 degrees of further curve progression.

Table 2 summarizes the seven studies reporting curve progression > 5 degrees 2–4 years after skeletal maturity for full-time bracing. The range of curves progressing > 5 degrees after stopping brace treatment ranged from 0 to 52%. Cobb angle progressed by 5 degrees or more in 236 of 700 patients (33.7%). The studies had different indications for stopping bracing and it was often not possible to determine whether progression was after commencing weaning or completely stopping bracing.

**Table 2:** Studies reporting percentage of patients with curve progression > 5 degrees 2–4 years after stopping brace treatment in AIS patients (full-time rigid braces only)

Paper	Bracing inclusion*	n	Cobb angle start bracing	Cobb angle at weaning	Indication weaning	Weaning method	Follow-up	Number progressing > 5 degrees (%)	Comments
Aulisa et al. 2009	CA 25–40 Female only	50	29.3 ± 5.2	-	Ring apophysis fusion on lateral radiograph	2–4 h reduction every 4 months	2 years	0/50 (0%)	
Zaina et al. 2009	Not stated	68	26.8	21.8	Risser 3	Gradual reduction 2–3 h/day until night only at 6 months	2.7 years	16/68 (23.5%)	Exercises during weaning
Guo et al. 2014	CA 20–40 Female only	17	24.0	21.8	Risser stage 4 and more than 2 years post-men and no growth between two visits	Not stated	4 years (2–6.4)	5/17 (29.4%)	
Shi et al. 2016	CA 20–40 Female only	200	27.7 ± 5.9	30.1 ± 10.4	Risser stage 4 and more than 2 years post-men and no growth between two visits	Night wearing for 6 months	1 year + 2 years	60/200 (30%) 1 year 93/200 (46.5%) 2 years	
Cheung et al. 2019	CA 25–40 < 1 year post-men	144	32 ± 5	35.5 ± 7.3	Risser Stage 4, no growth in stand/sit height/arm span in 6 months, at least 2 years post-men	Stop + discarded	2 years + (mean 3 years)	42/144 (29.2%)	All stopped using brace at least 24 h before X-ray
Grothaus et al. 2020	CA 20–40 Female only	42	33 ± 9	-	Risser 4; no height change	Stop	2 years	22/42 (52.4%)	
Cheung & Cheung 2021	CA 25–40 < 1 year post-men	179	-	34.6 ± 7.7	Risser Stage 4, no growth in stand/sit height/arm span in 6 months, at least 2 years post-men	Not stated but usual in the unit to stop and discard	2 years	58/179 (32.4%)	Different cohort from 2019 study All stopped using brace at least 24 h before X-ray
Summary								236/700 (33.7%)**	

^*^Bracing inclusion: AIS, age 10–14, Risser 0–2 (unless stated)

^**^2-year results used when 1- and 2-year follow-up given

Men = menarche

### Factors affecting curve progression after discontinuing brace treatment

Papers specifically evaluating curve progression and discontinuing bracing have suggested:

- Three studies have reported higher Cobb angles at brace weaning increase the risk of further curve progression with curves 40 degrees or larger possibly being at higher risk and obviously being closer to a surgical threshold [22, 26, 34].
- Vertebral rotation of 20 degrees or more has a three-fold increase in the chance of curve progression by more than 5 degrees [35].
- Stable curves less than 25 degrees at weaning could be considered suitable for early weaning (bone age 13.9 years) [21].
- Scoliosis-specific exercises performed during brace weaning may reduce the risk of curve progression [25].

### Gap analysis

Eighteen of the included articles specifically made recommendations for future research: usually, the need for prospective studies, randomized controlled trials, longer follow–up, or validation of findings in other populations. Two studies indicated that the predictors of curve progression after brace treatment from their work were unclear and more robust predictors needed to be found. One article specifically mentioned the lack of any published comparison of the effectiveness of different braces in the treatment of thoracic curves according to SRS and SOSORT criteria, suggesting that a meta-analysis was needed [33]. Another recent study suggested that the definition of treatment success should be reconsidered [26].

## Discussion

### Study characteristics

There was a fairly even number of articles published each year; over half were published in Europe (*n* = 24) and a significant number in Asia (*n* = 10) and North America (*n* = 9). Only three articles had the same aims as those of this review, with the most common study aim being to evaluate a specific type of brace and its effectiveness in treating adolescent idiopathic scoliosis (*n* = 16). Most studies follow the SRS and SOSORT criteria for bracing [4, 8, 36] (AIS, age 10–15 years, Risser 0–2, Cobb angle 25–40 degrees), extended slightly by the BrAIST study [37] to include curves 20–40 degrees. Some do not report these criteria, making it difficult to interpret analyses across studies.

### When to discontinue brace treatment?

There were three studies with the same aims as this review. One such study suggests that Risser staging as a criterion for stopping brace treatment is inadequate, and that bone age measurement using Sanders’ stages (SS) or distal radius and ulnar (DRU) classification is more accurate, with weaning starting at Sanders’ stage 8 and radius grade 10/ulnar grade 9 being the earliest and most protective time point [34]. The same author reports in a later article using SS7 as a criterion for stopping brace treatment, particularly in curves of less than 40 degrees. Instead, the DRU classification suggests using ulnar 8 as the criterion for stopping brace treatment, and waiting until the distal radius and ulnar have reached complete fusion (Sanders’ 8) is not necessary [27]. In contrast, another article suggests that there is no significant difference in curve progression between the group who stopped bracing before SS7 and the group who stopped bracing after SS7 [26]. Also, curve progression may lag behind growth [38]. In summary, these current studies do not provide a sound recommendation on the criteria for when to stop brace therapy.

The bracing best practice guideline produced by Roye et al. (2020) recommended “Sanders’ stage, Risser stage, change in height, curve magnitude, and curve progression should be considered when discontinuing bracing.” The aim of bracing is probably to reach 2 years after skeletal maturity with the smallest curve possible and to avoid the curve reaching the most commonly used surgical threshold of 50 degrees. Clearly early removal from brace is likely to increase the chance of curve progression, while prolonged bracing may increase the known psychological issues [7, 39]. With at least one confounding factor of curve size, identifying the optimal timing of brace cessation will require a large cohort study collecting all the candidate factors: Risser stage; height change; time from menarche; hand and wrist X-rays for skeletal age; distal radius, ulnar stages, and Sanders’ stage. It is likely that a combination of factors will produce the best prediction of when to stop bracing.

### How to discontinue brace treatment?

Roye et al. (2020) recommended “Once the decision to stop bracing has been made, there should be a weaning period of at least 6 months before fully discontinuing the brace.” While weaning is commonly performed, there is no scientific evidence to show the benefit of weaning over discontinuation of bracing at any given definition of skeletal maturity. Clearly the questions of when and how to discontinue brace treatment are closely linked.

### What happens to the spine curve after brace treatment is stopped?

The results show the limited evidence for curve progression after bracing, with those studies reporting this having variable indications for stopping brace treatment, weaning protocols, and length of follow-up. Ideally, studies should report the mean degrees of curve progression and the number (percent) of patients progressing more than 5 degrees after brace treatment is stopped.

It is important to distinguish curve progression in the first few years after brace weaning from progression over many years of adult life. From the available literature, early curve progression after brace treatment is a mean of 3.8 degrees, with approximately 34% progressing more than 5 degrees.

### Strengths and weaknesses of the review

This review included all pertinent literature to date with no restrictions on publication date or country. This broadens the extent of coverage of the review and strengthens the results. As the focus of a scoping review is not on quality or risk assessment of included articles, this allows a full description of all included studies in relation to the review aims.

However, because no restrictions were put on language of articles, there were some articles (*n* = 12) that could not be found as English copies, or where English translations were not available. As this work was unfunded, it was not possible to obtain translations. It is possible that these articles included information helpful to this review that is different from those articles that were included. Future reviews could consider including articles in all languages to improve the accuracy of the review. The same applies to the four articles that could not be obtained as full-text copies.

In line with PRISMA-ScR [10], this review did not include an assessment of quality. While this made the reporting of the literature more comprehensive, the quality of the included articles was variable and heterogeneity between articles was high. A systematic review could be considered to improve the quality of available review evidence on this topic.

### Further research

This review highlighted the lack of agreement on timing and method of cessation of brace treatment in this patient population; comparisons between studies were difficult due to the heterogeneity of baseline data reported in included articles; and many articles had differing aims, meaning they did not report on all aspects of data this review focused on. Therefore, this review alone cannot provide definitive recommendations on when and how to discontinue bracing.

The most appropriate timing and method of brace cessation in adolescent idiopathic scoliosis remain uncertain, and future research could be focused on improving evidence for this. In line with the priorities for further research identified by some of the articles included in this review, it is recommended that prospective studies should be completed to provide more accurate recommendations. Large, prospective cohort studies, including registry-based multi-center studies, will improve knowledge on when and how to stop bracing and the risk factors associated with curve progression after brace treatment. A randomized controlled trial would ensure high-quality evidence, removing selection bias from patient samples and standardizing the baseline data to ensure comparable results. However, randomized controlled trials of when and how to discontinue brace treatment would not be straightforward given the vast array of options to determine both factors, as seen across the articles included in this review. When to wean could be evaluated by surveying potential recruiting spinal surgeons to determine an acceptable range to produce an “early” and “delayed” brace weaning time for a randomized controlled trial. In terms of weaning protocols, any future research should investigate the weaning choices patients might tolerate best after 2–4 years of full-time bracing. Stopping bracing would be one arm of a randomized controlled trial, and patients’ agreed-upon weaning protocols would be the other arm. This could be combined with the groups for timing of brace cessation as above, for a four-armed trial.

## Conclusions

This review does not allow us to make recommendations on the most appropriate timing and/or method of brace cessation in adolescent idiopathic scoliosis. From current evidence, it seems that progression after stopping brace treatment is a significant problem, with approximately 34% of patients showing progression by more than 5 degrees. Large curves seem more likely to progress. It is therefore recommended that prospective cohort and randomized controlled trials be conducted to investigate the timing and modalities of cessation of brace treatment. Brace inclusion criteria must be consistent and in keeping with SRS criteria although the BrAIST study has expanding the curve size to 20–40°. Radiographs at all time points should be out-of-brace except for documenting in-brace correction. A systematic review could be considered as a follow-up to this scoping review but would have issues from different inclusion criteria, different criteria for stopping bracing, different weaning criteria, and different lengths of follow-up after stopping bracing.

## Ethics approval

Ethics approval was not applicable for this scoping review.

### Electronic supplementary material

Below is the link to the electronic supplementary material.

Supplementary file1 (PDF 435 KB)

## Data Availability

Data sharing is not applicable to this article as no datasets were generated or analyzed during the current study.

## Appendix 1: Search Strategy

Ovid MEDLINE(R) and Epub Ahead of Print, In-Process, In-Data-Review and Other Non-Indexed Citations and Daily < 1946 to April 11, 2022 >

**Table:**

#	Search term	Number of results
1	*brace/	3319
2	*orthotic device/	4302
3	Brace.mp. or Braces/	9126
4	Bracing.mp	3924
5	1 or 2 or 3 or 4	15,036
6	*scoliosis/	16,551
7	Scoliosis.mp	27,750
8	6 or 7	27,750
9	Adolescent/ or adolescent.mp	2,206,706
10	Child/ or child.mp	2,272,204
11	9 or 10	3,455,520
12	5 and 8 and 11	1936
