# Supplementary material for: Discontinuation of brace treatment in adolescent idiopathic scoliosis (AIS): a scoping review
Source: Spine Deform. 2024 May 1;12(5):1217–28. doi: 10.1007/s43390-024-00882-3 (PMC11343946; doi:10.1007/s43390-024-00882-3)
Supplement: Supplementary file 1 — Supplementary file1 (PDF 435 KB) [file 43390_2024_882_MOESM1_ESM.pdf]

Study	Scope of study	Intervention(Brace cessation)			Follow-up			
**Author, year, country**	**Inclusion indications**<br>**1. Number with AIS:**<br>**(must be able to extract data on AIS patients separately)**<br>**2. Age at brace start:**<br>**(mean, SD, range)**<br>**3. Cobb angle at brace start :**<br>**(mean, SD, range)**<br>**4. Risser s:**	**Indication for cessation:**	**Method of cessation: (stop or wean, how this was done)**	**Cobb angle at brace cessation: (mean, SD, range)**	**Cobb angle at 1 year after brace cessation: (mean, SD)**	**Cobb angle at 2 years after brace cessation: (mean, SD)**	**Recommendations made**	**Additional relevant data**
**Aulisa 2017 Italy**	1. Number with AIS: 93<br>2. Age at brace start:<br>mean: 11.1 ± 2.4; range: 10-14<br>3. Cobb angle at brace start:<br>mean: 32.28 ± 9.4; range: 20° - 55°<br>4. Risser s: 0 - 2	when ring-apophysis fusion was seen on a laterolateral view radiograph	wean: 2 to 4h of bracing<br>reduction at 4 month intervals	mean: 19.4 ± 10.8	Cobb angle at 5 year after brace cessation:<br>mean: 20.7 ± 11.2	Cobb angle at 10 years minimum after brace cessation:<br>mean: 22.1 ± 12.1	The brace was effective for long-term treatment and there was a loss of correction but no excess of original curvature after long-term follow-up. the initial size of the Codd angle had no effect on the long-term outcome.	
**Bulthuis 2008 Netherlands**	1. Number with AIS: 63<br>2. Age at brace start:<br>mean: 11.3 ± 3.1;<br>3. Cobb angle at brace start:<br>mean: 30.2 ± 7.5;<br>4. Risser s: 0 - 1	When the radiographs showed Risser sign 4 or, for girls, 2 years postmenarche and patients did not show any further growth at length measurements.	wean: generally complete within 4 weeks (No specific method of weaning is stated.)	mean: 22.3 ± 6.4<br>mean correction: 20 ± 15%		mean correction: 19 ± 13%	Compared with published studies on the natural history of idiopathic scoliosis, use of the Triac brace appeared to significantly improve the course of curves between 20 and 40° in skeletally immature individuals.	
**Cheung 2019 China**	1. Number with AIS: 144<br>2. Age at brace start:<br>range: 10-14;<br>3. Cobb angle at brace start:<br>mean: 32 ± 5°<br>4. Risser s: 0 - 2	they were Risser Stage 4, had no growth in body height, sitting height, and arm span in the past 6 months of followup, and were at least 2 years postmenarche.	stop: Braces were discarded on the day of brace weaning to avoid further use	mean ± SD: 35.5 ± 7.3	Progression of the curve was determined as any >5° increase in Cobb's angle between measurements at any subsequent six-month follow-up. There was no provision for measurements to be taken at a fixed time.	mean degree of curve progression after weaning was 8.3 ± 3.0°	Brace weaning indications using Risser staging are inadequate. Regardless of the discontinuation of brace treatment at any skeletal maturity, patients with large curves will experience progression of curvature. Bone age measurement by either Sanders staging or the distal radius and ulna classification provides clearer guidelines for brace weaning, resulting in the least postweaning curve progression. Weaning in patients with Sanders Stage 8 and radius Grade 10/ulna Grade 9 provides the earliest and most protective timepoints for initiating brace weaning.	
**Cheung 2021 China**	1. Number with AIS: 179<br>2. Age at brace start: NA<br>3. Cobb angle at brace start: NA<br>4. Risser s: NA	At Risser stage ≥ 4, in those without any interval growth (no increase of standing height and arm span) compared with the visit at least six months previously, and two years post-menarche for girls.	wean: No specific method of weaning is stated	mean: 34.6°<br>SD: 7.7°	Curve change at six months after weaning vs at time of weaning:<br>mean° (SD): 2.0 (2.4)	Curve change at two-year follow-up vs at time of weaning:<br>mean° (SD): 3.9 (4.1)	The use of SS7b is recommended for brace weaning, especially for patients with curves < 40° at the time of weaning, to reduce the rate of curve progression after weaning. For clinicians using the DRU classification for the assessment of skeletal maturity, U8 is a good indicator for brace weaning. Reaching full fusion in both distal radial and ulnar physes (as at Sanders stage 8) is not necessary	Curve change at two years vs six months follow-up: mean° (SD): 2.4 (3.2)
**Shi 2016 China**	1. Number with AIS: 200<br>2. Age at brace start:<br>mean: 12.1 ± 1.2; rang: 10-14<br>3. Cobb angle at brace start:<br>mean: 27.7 ± 5.9; rang: 20-40<br>4. Risser s: 0 - 2	1) Risser stage >4 and more than 2 years post menarche and<br>2) no growth between 2 visits.	wean: the brace wearing time were shortened to night wearing for 6 more months	mean ± SD: 30.1 ± 10.4°	mean ± SD: 33.6 ± 10.7°<br>(at 6 months: mean ± SD: 32.7± 10.3°)	mean ± SD: 35.0 ± 11.2°<br>(at lase follow-up: mean ± SD: 35.6± 12.0°)	Curve progression after brace weaning is observed in 43.5% AIS patients. The highest risk occurs within 6 months after brace weaning whereas Cobb angle remains stable after 2 years' follow-up. High Cobb angle at brace weaning indicates high risk of curve progression after brace weaning. Curve magnitude becomes stable beyond 2 years after brace weaning.	

**Simony 2020 Denmark**	1. Number with AIS: 124<br>2. Age at brace start: (The mean age of all patients was not available but the mean age of the different groups was calculated according to the curvature classification at the beginning)<br>20°-29°: mean (SD): 13.16 (0.99);<br>30°-39°: mean (SD): 13.38 (1.21);<br>40°-45°: mean (SD): 13.54 (1.45);<br>3. Cobb angle at brace start: (There is no mean Cobb value for all patients, only the number of people in the different curvature classification groups)<br>20°-29°: 25; 30°-39°: 42; 40°-45°: 13;<br>4. Risser s: NA	2 years after menarcheal for the girls, and when no increase in standing height was measured in the boys in the 6-month interval between clinical examinations	stop	20°-29°: mean (SD): 22.04 (8.92);<br>30°-39°: mean (SD): 30.40 (7.69);<br>40°-45°: mean (SD): 41.54 (5.40);	The text mentions measurements at 6 and 12 months after cessation of treatment, respectively, but does not show these two data. Only the amount of change in Cobb values in the different groups and the number of patients in the different groups with a change of more than 5 degrees are available.	Change cobb:<br>20°-29°: mean (SD): 3.20 (8.24);<br>30°-39°: mean (SD): 2.79 (6.77);<br>40°-45°: mean (SD): −0.38 (4.93);<br>Progression>5°: each group has three patients	Providence nighttime braces are an efective treatment for adolescent idiopathic scoliosis patients. This study reports a success rate of 89%, and the results are comparable to full-time treatment with the Boston brace. In-brace correction is crucial in part-time bracing, and we recommend at least 70% curve correction, if part-time bracing should be considered. Close attention is needed during treatment of patients with a Harrington factor>6° or an inbrace correction<60%, since this might be associated with a high risk of progression during PNB treatment.	mean in-brace correction was 83% (57–100%)
**Steen 2015 Norway**	1. Number with AIS: A total of 381 patients were included in the study, including 351 patients with AIS.<br>2. Age at brace start:<br>The mean age of all patients was not available and was divided into Unplanned early weaning, Planned early weaning and Ordinary bracing groups with mean ages of: 11.5 (7.7–15.5); 11.4 (7.5–16.0) and 13.7 (6.9–17.1) respectively<br>3. Cobb angle at brace start:<br>mean: 33.1°; range: 20°–57°<br>4. Risser s: <3	Ordinary bracing groups: Risser 5 in boys or Risser 4 or two years after menarche in girls<br>Planned early weaning: patients demonstrated a good effect of the brace with a stable reduction of the primary curve to 25° or less at repeated measurements.	wean: during a period of 2–3 months by 2 h less bracing time every week	Unplanned early weaning: 35.0 (6–58);<br>Planned early weaning: 20.6 (1–45);<br>Ordinary bracing: 29.3 (6–58);	Only the curvature values at the last follow-up visit are available.<br>Unplanned early weaning: 34.8 (13–62) ;<br>Planned early weaning: 25.1 (9–54) ;<br>Ordinary bracing: 34.0 (7–81);		Planned early weaning reduces the time to correction and has good clinical results. This procedure may be attempted if curve reduction is stable over time and the primary curve is 25° or less in patients with several years of expected bracing. The patients should be monitored carefully and regularly at 4-6 months intervals until maturity, and a new brace should be prepared if the curve increases significantly.	
**Zaina 2009 Italy**	1. Number with AIS: 68<br>2. Age at brace start: NA<br>3. Cobb angle at brace start: There are no averages for all patients, and they are divided into four groups according to the exercise performed during weaning.<br>SEAS: 25.4° ± 8.9; OTH: 26.8° ± 6.5;<br>DIS: 29.4° ± 8.9; NO: 23.6° ± 8.9<br>4. Risser s: NA	Start of brace weaning was defined as the first visit during which it was prescribed that a brace be worn for less than 18/24 hours. At this stage an initial x-ray without the brace within six hours was made and, weaning started if Risser 3 stage was reached despite the patient's age.	wean: all the patients gradually reduced the daily hours of brace usage by two to three hours (according to clinical and/or radiographic evaluations) over six-month intervals until the prescription reached eight hours nightly, and then stopped after six months.	mean: 22 ± 8°<br>SEAS: 21.3° ± 10.4; OTH: 22.9° ± 6.8;<br>DIS: 22.1° ± 9.7; NO: 19.2° ± 5.2	Only the curvature after 2.7 years was recorded<br>SEAS: 21.6° ± 9.4; OTH: 22.0° ± 6.9;<br>DIS: 26.1° ± 9.7; NO: 22.3° ± 7.3		Exercises can help reduce the correction loss in brace weaning for AIS. Especially with continuous exercise.	Percentage of Cobb angles deterioration:<br>SEAS: 14.3%; OTH: 20%;<br>DIS: 28%; NO: 40%;
**Allington 1996 United States**	1. Number with AIS: 188<br>2. Age at brace start: NA (At least 9 years old)<br>3. Cobb angle at brace start:<br>LESS THAN THIRTY DEGREES:<br>Full-Time Bracing: 25 rang: 20-29<br>Part-Time Bracing: 24 rang: 20-28<br>Electrical Stimulation: 25 rang: 20-29<br>THIRTY TO FORTY DEGREES:<br>Full-Time Bracing: 35 rang: 30-40<br>Part-Time Bracing: 34 rang: 30-40<br>Electrical Stimulation: 33 rang: 30-40<br>4. Risser s: 0 - 1	When the girls had a Risser sign of 4 and were two years postmenarche and when the boys had a Risser sign of 5.	wean: took a mean of one year (no specific weaning method is stated.)	LESS THAN THIRTY DEGREES: 24<br>THIRTY TO FORTY DEGREES: 36	Only the curvature of the last follow-up was recorded.<br>LESS THAN THIRTY DEGREES:<br>Full-Time Bracing: 28<br>Part-Time Bracing: 28<br>Electrical Stimulation: 35<br>THIRTY TO FORTY DEGREES:<br>Full-Time Bracing: 41<br>Part-Time Bracing: 41<br>Electrical Stimulation: 43		Full-time and part-time bracing were equally effective in preventing progression of curves of 40 degrees or less in skeletally immature patients who had adolescent idiopathic scoliosis.	

**Andersen 2002 Denmark**	1. Number with AIS: 136<br>2. Age at brace start:<br>median: 14.1 (range 10–19)<br>3. Cobb angle at brace start: Greater than 30°<br>4. Risser s: less than 3	Over a 6-month period, was initiated when the patient's curve had not progressed between two visits after taking into account the patient's skeletal maturity as guided by the Risser sign.	wean: no specific weaning method is stated.	NA	No changes in Cobb values were recorded.		Weaning should be initiated at the latest 3 years after menarche. Not hesitate to stop the brace treatment if we find a patient to be vulnerable.	24% of those patients who had started weaning after the age of 16 years felt reserved in their relations with the other sex, compared to 7% of the patients who had started weaning before the age of 16 years. The percentage of patients that felt reserved towards the opposite sex increased with sexual maturity, expressed as age relative to menarche, at time of weaning from the brace.
**Appelgren 1990 Sweden**	1. Number with AIS: 121<br>2. Age at brace start: NA<br>3. Cobb angle at brace start:<br>mean curves proximally were 32 ± 8° and distally 27 ± 9°<br>4. Risser s: NA	NA	wean: no specific weaning method is stated.	mean curves proximally were 22 ± 9° and distally 19 ± 9°	mean curves proximally were 28 ± 10° and distally 25 ± 11°	mean curves proximally were 30 ± 10° and distally 26 ± 11°	The correction of the thoracic and lumbar curves by Boston brace treatment is caused by a decteased tilt of EVA B. This cannot be shown by the Cobb method. The EVA method also makes it possible to analyze the scolioticcurve in greater detail. By studying the range of asymmetry one seems to be able to predict the results of the brace treatment of the thoracic curve, in S-shaped scolioses, with right convexities of the thoracic curves, and left convexities of the lumbar curves.	
**Aulisa 2014 Italy**	1. Number with AIS: 395<br>2. Age at brace start:<br>mean: 12.6<br>3. Cobb angle at brace start:<br>mean: 30.5; SD: 8.0<br>4. Risser s: 0 - 2	Weaning was started when ring-apophysis fusion was seen to begin on a latero-lateral (LL) radiograph view, which corresponds to a Risser sign 4 or 5 on an antero-posterior (AP) standing radiograph view.	wean: 2 to 4 hours bracing reduction at 2-month intervals.	NA	Only the exact Cobb value at the last follow-up visit is provided.<br>mean: 13.1; SD: 10.0		Curve progression and referral to surgery are lower in patients with high brace compliance. Bracing discontinuation up to 1 month does not impact on the treatment outcome. Conversely, wearing the brace only overnight is associated with a high rate of curve progression.	This study was grouped according to different adherence.
**Aulisa 2015 Italy**	1. Number with AIS: 102<br>2. Age at brace start:<br>mean: 11.62 ± 1.1<br>3. Cobb angle at brace start:<br>mean: 31.51 ± 4.34 SD<br>4. Risser s: 0 - 2	When the ring-apophysis fusion was complete on a LL X-ray	wean: no specific weaning method is stated.	mean: 16.6 ± 9.0		mean: 20 ± 7.6	The Lyon brace, through its biomechanical action on vertebral modeling, is highly effective in correcting thoracic curves in particularly when the SOSORT guidelines were adopted in addition to the SRS criteria.	End of treatment<br>mean: 16.3 ± 9.6
**Aulisa 2009 Italy**	1. Number with AIS: 50<br>2. Age at brace start:<br>mean: 11.8 ± 0.5<br>3. Cobb angle at brace start:<br>mean: 29.30 ± 5.16 SD; rang: 25-40<br>4. Risser s: 0 - 2	When<br>ring apophyses fusion was complete on a latero-lateral X-ray.	wean: no specific weaning method is stated.	NA		mean: 14.67 ± 7.56	The PASB, due to its peculiar biomechanical action on vertebral modelling, is highly effective in correcting thoraco-lumbar curves.	
**Aulisa 2012 Italy**	1. Number with AIS: 40<br>2. Age at brace start:<br>mean ± SD: 11.6 ± 0.7 year<br>3. Cobb angle at brace start:<br>mean ± SD: 26.4 ° ± 2.8<br>4. Risser s: 0 - 2	When ring-apophysis fusion was seen to begin on a laterolateral view radiograph.	wean: 2 to 4 hours bracing reduction at 4-month intervals.	Beginning of Weaning:<br>mean ± SD: 8.4 ± 5.2<br>End of Treatment:<br>mean ± SD: 11.6 ± 7.7		mean ± SD: 13.8 ° ± 7.9	The PASB allows a complete curve correction in most cases. No patients exhibited curve progression.	

**Aulisa 2020 Italy**	1. Number with AIS: 163<br>2. Age at brace start:<br>rang: 10–17 years<br>3. Cobb angle at brace start:<br>mean: 28.98 (±7.9); range 20°- 60°<br>4. Risser s: 0 - 4	When ring-apophysis fusion was seen on a lateral view radiograph.	wean: 2 to 4 hours bracing reduction at 4-month intervals.	End of Treatment:<br>mean ± SD: 13.88 ± 10.8		mean ± SD: 15.35 ° ± 11.68	The PASB brace is effective for the treatment of lumbar and thoracolumbar scoliosis and is characterized by positive long-term outcomes, including in patients demonstrating moderate curves. In both groups, at 10-years minimum follow-up after bracing, scoliotic curves did not deteriorate beyond their original curve size after bracing in both groups at the 10-years minimum follow-ups.	
**Bassett 1986 United States**	1. Number with AIS: 79<br>2. Age at brace start:<br>mean: twelve years and five months;<br>rang: nine years and four months to fifteen years and five months<br>3. Cobb angle at brace start:<br>Separated the patients into 3 groups according to their curve pattern:<br>(1) thoracic: mean: 29; rang: 20-39<br>(2) thoracolumbar-lumbar: mean: 25; rang: 20-38<br>(3) double major: mean: 30; rang: 20-39<br>4. Risser s: 0 - 1	When iliac apophysis is shown to be fully capped (Risser grade 4) on radiographs.	wean: warning the brace only 12 hours a day over the next year, with continued evaluation for changes in stability of the curve. Advised patient to continue wearing the brace at night for an additional 1 to 2 years. The average weaning phase was 2 years (rang: 2 months to 4 years and 6 months).	Bear correction in brace:<br>(1) thoracic: mean: 14; rang: 0-35<br>(2) thoracolumbar-lumbar: mean: 12; rang: 2-33<br>(3) double major: mean: 18; rang: 2-28<br>Curve at end of weaning:<br>(1) thoracic: mean: 29; rang: 10-60<br>(2) thoracolumbar-lumbar: mean: 22; rang: 7-40<br>(3) double major: mean: 31; rang: 8-49		curve at follow-up:<br>(1) thoracic: mean: 31; rang: 10-60<br>(2) thoracolumbar-lumbar: mean: 23; rang: 8-40<br>(3) double major: mean: 33; rang: 5-55	Wilmington brace favorably alters the natural history of 20 to 39-degree idiopathic curves.	
**Bohl 2014 United States**	1. Number with AIS: 34<br>2. Age at brace start:<br>mean: 12.9; rang: 10-15<br>3. Cobb angle at brace start:<br>mean: 27.2°±3.8° rang: 20°-36°<br>4. Risser s: 0 - 2	Bracing was continued until skeletal maturity as determined by Risser stage 4 or higher, no axial growth over 2 visits 6 months apart, or at least 18 months postmenses.	stop	NA		last follow-up:<br>mean: 36.6°±10.8°<br>rang: 17°-55°	This study suggests a rate of progression that is similar to or lower than rates reported in natural history studies, possibly supporting the efficacy of the Providence brace. Future prospective studies should include an objective assessment of compliance using a validated method such as a temperature sensor, which allows calculation of a dose response, where the dose is hours worn per day. In addition to simple measures of compliance, attention to the psychosocial effect of nighttime vs full-time bracing as a secondary outcome of a prospective trial would also be useful to clinicians.	Seventeen (50%) patients progressed more than 5°, and 9 (26%) progressed to more than 45° and had fusion surgery recommended or performed. Males had a higher rate of progression more than 5° than did females (100% vs 41%; P=.015).
**Brox 2012 Norway**	1. Number with AIS: 495<br>2. Age at brace start:<br>Divided into Compliers (n = 389) and Non-compliers (n = 106)<br>Compliers: mean(SD): 13.2 (1.9)<br>Non-compliers: mean(SD): 12.9 (2.1)<br>Divided into Attended (n = 381) Did not attend (n = 114)<br>Attended: mean(SD): 13.2 (1.9)<br>Did not attend: mean(SD): 13.0 (2.2)<br>3. Cobb angle at brace start:<br>Divided into Compliers (n = 389) and Non-compliers (n = 106)<br>Compliers: mean(SD): 33.1 (7.2)<br>Non-compliers: mean(SD): 32.8 (7.6)<br>Divided into Attended (n = 381) Did not attend (n = 114)<br>Attended: mean(SD): 33.1 (7.2)<br>Did not attend: mean(SD): 33.0 (7.7)<br>4. Risser s: 0 - 3	2 years after menarche or until Risser 4 or 5.	wean: no specific weaning method is stated.	Divided into Compliers (n = 389) and Non-compliers (n = 106)<br>Compliers: mean(SD): 26.4 (9.5)<br>Non-compliers: mean(SD): 33.5 (10.2)<br>Divided into Attended (n = 381) Did not attend (n = 114)<br>Attended: mean(SD): 28.4 (10.2)<br>Did not attend: mean(SD): 26.6 (10.0)	Divided into Compliers (n = 389) and Non-compliers (n = 106)<br>Compliers: mean(SD): 27.4 (9.2)<br>Non-compliers: mean(SD): 33.7 (10.4)<br>Divided into Attended (n = 381)<br>Did not attend (n = 114)<br>Attended: mean(SD): 28.9 (9.7)<br>Did not attend: mean(SD): 27.6 (9.9)	Divided into Compliers (n = 389) and Non-compliers (n = 106)<br>Compliers: mean(SD): 28.1 (9.2)<br>Non-compliers: mean(SD): 33.2 (9.9)<br>Divided into Attended (n = 381)<br>Did not attend (n = 114)<br>Attended: mean(SD): 29.4 (9.9)<br>Did not attend: mean(SD): 27.8 (9.6)	The risk for curve pro gression and surgery are reduced in patients with good brace compliance.	The number of patients who had surgery after weaning:<br>Divided into Compliers (n = 389) and Non-compliers (n = 106)<br>Compliers: 11/389; Non-compliers: 2/106

**Cheung 2020 China**	1. Number with AIS: 586<br>2. Age at brace start:<br>mean: 12.6 ± 1.2;<br>3. Cobb angle at brace start:<br>mean: 31 ± 4°<br>4. Risser s: 0 - 2	Age of 18 years or Risser Stage 4, no growth in body height for the past 6 months, and 2 years post-menarche	wean: follow a gradual weaning protocol over 6 months from the day of brace weaning.	NA		Divided into Improvement, Unchanged and Deterioration. Only the change in Cobb values after a mean of 2 ± 1 years of follow-up are mentioned.<br>Improvement: mean: 0 ± 4°<br>Unchanged: mean: 1 ± 3°<br>Deterioration: mean: 1 ± 4°	Curve regression occurs after underarm bracing and is associated with superior patient-reported outcome scores. This possible change in Cobb angle should be explained to patients before and during bracing. Whether this may help improve patients' duration of bracewear should be addressed in future studies. Patients with well-fitting braces may experience curve improvement and possible vertebral remodeling. Those braced at a younger age and with increased vertebral wedging are more likely to have curve progression.	The discussion section mentions that because studies have shown that curvature still develops after weaning using the current weaning criteria, the current criteria are inaccurate and further research is needed.
**Coillard 2003 Canada**	1. Number with AIS: 195 (only 71 patients had terminated treatment)<br>2. Age at brace start:<br>mean: 13 years; SD: 1 year<br>3. Cobb angle at brace start:<br>mean: 29°; SD: 8° (for those 71 patients: mean: 29°; SD: 7°)<br>4. Risser s: 0 - 3	Near skeletal maturity, or after 2 years of regular menstruation.	stop	at the end of treatment:<br>mean: 21°; SD: 12°	mean: 25°; SD: 11°	mean: 24°; SD: 11°	This initial cohort of idiopathic scoliosis patients who were treated with the SpineCor system reveals a positive treatment outcome at 2 years follow-up. This is reflected through a cumulative probability of success, which increases during treatment, and is maintained through 1 and 2 years follow-up.	At the last available visit, there were 109 patients still under treatment, with a mean treatment time of 1.5 years (SD 1 year).
**Coillard 2007 Canada**	1. Number with AIS: 170 (only 47 patients had 2 years of postbracing follow-up)<br>2. Age at brace start:<br>range: 10-15<br>3. Cobb angle at brace start:<br>for 47 patients group: 25-29 degrees: 21; 30-40 degrees: 26<br>for all 170 patient: 25-29 degrees: 67; 30-40 degrees: 103<br>4. Risser s: 0 - 2	At skeletal maturity (at least Risser 4)	stop	NA		The treatment success rate at 2 years was 95.7% (n = 47);<br>Forty of 47 patients stabilized their Cobb angle, and 5 patients still improved from the time the brace was discontinued up to 2 years of follow-up.	The SpineCor Brace is effective for the treatment of AIS. Moreover, the positive outcomes are maintained up to 2 years of follow-up beyond skeletal maturity. This particular feature of the SpineCor brace makes it very different to the already published literature on brace, in which apparent correction obtained during treatment can be expected to be lost over time. Future studies that will support and reinforce this finding are necessary.	39 immature patients (22.9%) from a total of 170 required surgical fusion while receiving treatment. The average curve magnitude at bracing in this particular group was 34 ± 5.1 degrees (range, 25-40 degrees).
**Danielsson 2007 Sweden**	1. Number with AIS: 106 (92 patients completed follow-up)<br>2. Age at brace start:<br>92 patients completed follow-up:<br>mean (SD): 13.7 (1.1) rang: 10.3–16.1<br>The 92 patients were further divided into 4 groups according to treatment<br>Brace Treatment From Start (n=35):<br>mean (SD):13.5 (1.4) rang: 10.3–16.1<br>Observation Only (n=40):<br>mean (SD): 14.0 (0.9) rang: 11.4–15.6<br>Observation/Braced(braced after increase ≥ 6°) (n=11):<br>mean (SD): 13.1 (1.0) rang: 11.3–14.4<br>Observation/Surgery (surgically treated after increase ≥ 6°)(n=6)<br>mean (SD): 13.5 (0.9) rang: 12.5–15.0<br>3. Cobb angle at brace start:<br>92 patients completed follow-up:<br>mean (SD): 30.2 (3.2) rang: 23–39<br>The 92 patients were further divided into 4 groups according to treatment<br>Brace Treatment From Start (n=35):<br>mean (SD):31.6 (3.1) rang: 26–38<br>Observation Only (n=40):<br>mean (SD): 29.2 (2.9) rang: 23–35<br>Observation/Braced(braced after increase ≥ 6°) (n=11):<br>mean (SD): 28.1 (1.9) rang: 25–32<br>Observation/Surgery (surgically treated after increase ≥ 6°)(n=6)<br>mean (SD): 32.8 (3.9) rang: 27–39<br>4. Risser s: 0 - 4	Until skeletal maturity. A skeletal age of 16 years or a chronologic age of 16 years in combination with a Risser sign of 4 or 5 were required to determine maturity.	NA	At completed treatment<br>92 patients completed follow-up:<br>mean (SD): 28.4 (6.4) rang: 10–42<br>The 92 patients were further divided into 4 groups according to treatment<br>Brace Treatment From Start (n=35):<br>mean (SD): 26.2 (5.8) rang: 14–37<br>Observation Only (n=40):<br>mean (SD): 30.6 (5.0) rang: 21–42<br>Observation/Braced(braced after increase ≥ 6°) (n=11):<br>mean (SD): 32.3 (6.4) rang: 20–42<br>Observation/Surgery (surgically treated after increase ≥ 6°)(n=6)<br>mean (SD): 19.8 (7.5) rang: 10–30		At present follow-up:<br>The 92 patients were further divided into 4 groups according to treatment<br>Brace Treatment From Start (n=35):<br>mean (SD): 31.9 (7.3) rang: 19–48<br>Observation Only (n=40):<br>mean (SD): 35.0 (6.5) rang: 21–48<br>Observation/Braced(braced after increase ≥ 6°) (n=11):<br>mean (SD): 39.4 (4.3) rang: 33–46	The curves of patients with AIS with a moderate or smaller size at maturity did not deteriorate beyond their original curve size at the 16-year follow-up. No patients treated primarily with a brace went on to undergo surgery, whereas 6 patients (10%) in the observation group required surgery during adolescence compared with none after maturity. Curve progression was related to immaturity.	None of the 41 patients who were treated with a brace from the start experienced a curve increase of ≥ 6°. In the 65 patients observed from the start, the curves of 26 patients (40%) increased by ≥ 6°; 13 patients received brace treatment and 6 underwent surgery due to curve size and residual growth.

**Fernandez-Feliberti 1995 United States**	1. Number with AIS: 101 (Receiving brace treatment: 54: without brace treatment: 47)<br>2. Age at brace start: range: 8-15<br>not treated group: ≤13: 18 (38.3%); >13: 29 (61.7%)<br>treated group: ≤13: 29 (53.3%); >13: 25 (46.3%)<br>3. Cobb angle at brace start:<br>observation group: ＜30°(8-15 years patients) 30-45°(skeletally mature patient and no evidence of progression in the past 6 months)<br>bracing group: 30-40° and patients with documented progression in a flexible curve ≤30°<br>not treated group: ≤29: 27 (57.45%); ≥30: 20 (42.55%)<br>treated group: ≤29: 25 (46.30%); ≥30: 29 (53.70%)<br>4. Risser s: NA	At the time of skeletal maturity, if there was no evidence of recent significant progression of the curve.	wean: gradual 6-month 'weaning' process	NA		final cobb:<br>not treated:<br>≤39: 28 (59.57%); ≥40: 19 (40.43%)<br>treated group:<br>≤39: 38 (70.37%); ≥40: 16 (29.63%)	The study shows how the TLSO bracing reduces the probability of curve progression. Children who are young (≤13 years) and who have an initial Cobb's angle of ≥30° obtained fewer benefits from bracing.	
**Green 1986 United States**	1. Number with AIS: 44<br>2. Age at brace start:<br>mean: 13; range: 9 years and 9 months to 15<br>3. Cobb angle at brace start:<br>mean: 31; range: 23° - 49°<br>4. Risser s: NA	At the end of skeletal growth, as determined by a radiograph showing bone age and also by completion of the excursion of the iliac apophyses.	stop	There were 25 patients whose information was not recorded in the form. The cobb ranges for the patients recorded in the table are: 16 to 48		At last follow-up:<br>mean: 30<br>rang: 15-51	Part-time wear of a brace can prevent progression of adolescent idiopathic scoliosis and can even afford some correction. Since it has been shown that small curves do not progress after skeletal maturity, the prevention of progression of a curve during adolescence should produce a good lasting result.	Two patients underwent surgical treatment and 19 patients had a change in curve of more than 5 degrees from the start of the brace to the last follow-up.
**Grothaus 2020 United States**	1. Number with AIS: 89<br>2. Age at brace start:<br>mean (SD): 13.8 (1.1)<br>3. Cobb angle at brace start: (Bracing was neither an exclusion nor inclusion criterion. The mean of all patients using the brace was not recorded, only the mean of all patients at SS7. Only rang of patients using brace is 10-20°)<br>mean (SD): 33° (9)<br>4. Risser s: 0 - 2	Risser 4	stop	NA		mean (SD): 38° (11)	A curve>40 degrees at SS7 is at high risk for progressing to a curve measuring >50 degrees or requiring surgery. Those with curves below this threshold still have potential to make clinically significant progression after skeletal maturity. Follow-up of patients beyond SS7 is essential for curves measuring>40 degrees. Reaching SS7 with a curve<50 degrees may not be the endpoint for curve progression, even if predictive of the end of spinal growth. It is therefore recommended that utilizing SS7 in conjunction with other measures of growth and maturity to guide treatment decisions.	There were 19 patients with curves ≥40 degrees at SS7; of which 11 (58%) progressed to >50 degrees or had surgery.Receiver operating characteristic curve analysis identified a threshold of 39.5 degrees curvature at SS7 associated with progression to >50 degrees or surgery. There were no patients with initial SS7 curves measuring ≤39.5 degrees who progressed past 50 degrees. There was no significant difference in the change in curve between the group that discontinued brace treatment before SS7 and the group that discontinued brace treatment after SS7. SS7 is not predictive of cessation of curve progression, even if it signals the end of spine growth.
**Guo 2014 China**	1. Number with AIS: 43 (5 patients were lost to follow-up and only 38 patients completed the study)<br>2. Age at brace start:<br>There are two groups depending on the brace used:<br>R group (n = 18): 12.6 ± 0.8 (10.9 to 13.8)<br>S group (n = 20): 12.3 ± 0.9 (10.4 to 13.7)<br>3. Cobb angle at brace start:<br>There are two groups depending on the brace used:<br>R group (n = 18): 24.0 ± 2.8 (20 to 30)<br>S group (n = 20): 24.1 ± 2.9 (20 to 30)<br>4. Risser s: 0 - 2	At the time of skeletal maturity which was defined by＜1 cm change in standing height made on two consecutive visits with 6 months apart, when Risser 4 was present and when the patient was 2-year post-menarche.	wean: No specific method of weaning is stated	Only values for patients who progressed beyond skeletal maturity and those who did not in the R-stable and S-stable groups are shown.<br>R-stable:<br>Progressed (n = 5): 21.2 ± 7.1 (15 to 33)<br>Stable (n = 12): 22.0 ± 5.4 (15 to 30)<br>S-stable:<br>Progressed (n = 5): 22.0 ± 2.7 (19 to 26)<br>Stable (n = 8): 21.5 ± 5.8 (13 to 28)		Further curve progression of > 5° beyond skeletal maturity:<br>R-stable group (n=17): 5 (29.4 %)<br>S-stable group (n=13): 5 (38.5 %)	Curve progression rate was found to be significantly higher in the SpineCor group when compared with the rigid brace group. Changing to rigid bracing could control further curve progression for majority of patients who previously failed with SpineCor bracing. For both SpineCor and rigid brace treatments, 30–40 % of patients who were originally successfully treated by bracing would exhibit further curve progression beyond skeletal maturity. The post-maturity progression rate was found to be 1.5 per year in the current study, which was relatively greater than those reported before.	
**Kawasaki 2021 Japan**	1. Number with AIS: 133<br>2. Age at brace start:<br>In-brace deterioration n=95 (71.4%) :<br>median: 144.0; mean ± SD: 145.8 ± 12.9 months<br>Out-of-brace deterioration n=38 (28.6%)<br>median: 147.0; mean ± SD: 148.8 ± 11.7 months<br>3. Cobb angle at brace start:<br>In-brace deterioration n=95 (71.4%) :<br>median: 32.0; mean ± SD: 32.0 ± 4.3°<br>Out-of-brace deterioration n=38 (28.6%)<br>median: 31.3; mean ± SD: 31.5 ± 3.7°<br>4. Risser s: 0 - 2	The indication for brace weaning was Risser stage≥4, no change in body height within 6 months, and 2 years post-menarche for females.	wean: Gradually weaned over the course of 6 months. The first 2 months with 8 hours reduction during the day, followed by only night wear in the next 2 months, and finally completely off in the final 2 months.	Initial in-brace Cobb:<br>In-brace deterioration n=95 (71.4%) :<br>median: 21.2; mean ± SD: 21.1 ± 6.3°<br>Out-of-brace deterioration n=38 (28.6%)<br>median: 22.9; mean ± SD: 23.5 ± 6.5°		The Cobb angle outcomes:<br>In-brace deterioration n=95 (71.4%) :<br>mean: 50.5 ± 10.3°<br>Out-of-brace deterioration n=38 (28.6%)<br>mean: 45.9 ± 9.2°	Patients may develop curve progression despite good correction on in-brace radiographs. Those with higher flexibility and suboptimal brace fitting are at-risk. In-brace and out-of-brace radiographs should be taken alternately for brace treatment follow-up.	Correction rate (%):<br>In-brace deterioration n=95 (71.4%) :<br>median: 32.7; mean ± SD: 34.2 ± 16.5<br>Out-of-brace deterioration n=38 (28.6%)<br>median: 25.0; mean ± SD: 25.3 ± 17.6

**Kenanidis 2021 Greece**	1. Number with AIS: 77<br>2. Age at brace start:<br>mean: 13.2±1.6; rang: 8–16<br>3. Cobb angle at brace start:<br>mean: 28.2±8.7; range: 12° - 60°<br>4. Risser s: 0 - 3	On skeletal maturation:<br>(1) Risser sign≥4<br>(2) 16 years old for females and<br>18 years old for males<br>(3) 3 years post-menarche for females.	stop	mean: 21.5±11.8<br>rang: 5–66		At 25.1 years after brace cessation:<br>mean: 25.4±13.8<br>rang: 4–76	A scoliotic curve is expected to lose some correction after bracing. The apical vertebral rotation post-bracing mainly afected the long-term curve behaviour. Adolescents with apical vertebral rotation greater than 20° after bracing may need further attention.	
**Lange 2011 Norway**	1. Number with AIS: 214 (there were 272 patients, with the remaining 58 patients with late-onset juvenile)<br>2. Age at brace start:<br>mean: 13.8 (1.3) years<br>3. Cobb angle at brace start:<br>mean: 33.3 (6.9)°<br>4. Risser s: < 4	2 years after menarche or at Risser sign 5, in some patients at Risser sign 4.	wean: No specific method of weaning is stated	mean: 29.0 (10.0)°		mean: 33.0 (12.7)°	Long-term results were satisfactory in most braced patients and similar in late-onset juvenile and idiopathic adolescent scoliosis. HRQL was slightly decreased in these patients, SRS-22 scores for self-image was significantly lower in both groups. Self-report indicates that scoliosis does not affect pregnancy and delivery, and that most patients are expected to work and have HRQL in the normal range at long-term.	
**Misterska 2017 Poland**	1. Number with AIS: 30<br>2. Age at brace start:<br>Mean (SD): 12.43 (1.83); rang: 10–14 years<br>3. Cobb angle at brace start:<br>Mean (SD): 32.2 (5.59); rang: 20–40<br>4. Risser s: 0-2	Risser sign 4 and minimum two years post-menarche (Risser Grade IV was identified after completing treatment in all patients involved in the study (100%))	NA	after treatment:<br>mean (SD): 37.87 (12.75)<br>rang: 10–70		at follow up:<br>mean (SD): 45.03 (17.41)<br>rang: 10–97	This retrospective examination of the function of the lumbar and cervical spine, in a highly specific subset of patients, revealed that persons with scoliosis treated in adolescence with a Milwaukee brace display significant limitations in everyday activities, due to LBP- and neckrelated impairment. In addition, back pain is associated with curve progression in a long follow-up after conservative treatment. Moreover, LBP-related disability coexists with restrictions experienced due to neck pain.	
**Montgomery 1990 Sweden**	1. Number with AIS: 168<br>2. Age at brace start:<br>mean: 14.3 ± 1.6;<br>3. Cobb angle at brace start:<br>mean: 33.2 ± 6.5;<br>4. Risser s: NA	NA	wean: No specific method of weaning is stated	at end of weaning from brace:<br>mean: 28.0 ± 10.7		at 2 years: mean: 32.5 ± 10.5<br>at last follow-up: mean: 33.1 ± 11.5	Brace treatment does not postpone curve progression to the late twenties; thus, there is no ground for the statement that surgical interventions will occur later. And a follow-up 2 years after weaning is sufficient to predict the clinical result with great accuracy (97%).	
**Negrini 2009 Italy**	1. Number with AIS: 48<br>2. Age at brace start:<br>mean: 12.8 ± 1.6 years<br>3. Cobb angle at brace start:<br>mean: 30.4 ± 4.4°<br>4. Risser s: 0 - 2	Risser 3	wean: lasted an average 2.2 years until Risser 5. Reduced the wearing of the brace by no more than two or three hours every six months.	NA		SRS:<br>Unchanged (improved): 96% (59%)<br>Worsened: 4%<br>No patient progressed beyond 45°<br>Cobb degrees:<br>Average (SD): -7.3 (8.4)<br>ISICO:<br>96% of patients had minimal and 65% optimal results	Respecting also SOSORT management criteria and thus increasing compliance, the results of conservative treatment were much better than what had previously been reported in the literature using SRS criteria only.	ISICO:<br>In all analysis performed, gender or patients braced only did not show any difference with the total population. The results at 2 years follow-up were not different from those at the end of treatment.
**Ovadia 2012 Israel**	1. Number with AIS: 93<br>2. Age at brace start:<br>mean: 12.87; rang: 9–16<br>3. Cobb angle at brace start:<br>mean: 31.97°; rang: 22–54°<br>4. Risser s: 0 - 2	Risser 4–5, Tanner 4–5, less than 1 cm of height gain between outpatient visits at an interval of 6–8 months.	wean: brace wearing was reduced to 14–16 h for 6 months, then additional reduction to night time only (6–8 h) for 6 more months, after which brace treatment was completely terminated.	at end of treatment:<br>Success group: mean: 28.59° (n=79)<br>Failure group: mean: 55.93° (n=14)		mean: 28.97°	The treatment of mild to moderate AIS with the RSC brace provides excellent clinical results. Its added benefit is enabling a three-dimensional correction of a three-dimensional deformity. Pre-treatment high Risser score, low Cobb angles, and low ATRs are associated with treatment success.	Risser score at start of treatment:<br>Success group: mean: 1.19 ± 1.12 (n=79)<br>Failure group: mean: 0.28 ± 0.31 (n=14)<br>p=0.0009

**Pellios 2016<br>Greece**	1. Number with AIS: 77<br>2. Age at brace start:<br>part Time (18 hours a day): mean: 13.24 (±1.9)<br>full Time (23 hours a day): mean: 13.19 (±1.49)<br>3. Cobb angle at brace start:<br>mean: 28.25 (±8.73)<br>part Time (18 hours a day): mean: 28.71 (±9.02)<br>full Time (23 hours a day): mean: 27.86 (±8.58)<br>4. Risser s: < 4	At skeleton maturation, 3 years pass over menarche for the female patients or no deterioration of scoliotic curve for a period of 24 month follow-up. Skeletal maturity was defined as a Risser sign ≥ 4 or the age of 16 and 18 years for girls and boys respectively.	stop	at brace removal:<br>mean: 21.58 (±11.54)<br>part Time (18 hours a day): mean: 20.43 (±10.3)<br>full Time (23 hours a day): mean: 22.55 (±13.03)		25 years post bracing:<br>mean: 25.48 (±13.87)<br>part Time (18 hours a day):<br>mean: 24.37 (±12.4)<br>full Time (23 hours a day):<br>mean: 26.4 (±15.08)	This cohort demonstrated moderate loss of correction 25 years post successful bracing during adolescence. The mean cohort Cobb angle however remained stable 25 years post bracing. No difference in terms of long term results and progression between 18 and 23 h of self-reported bracing hours. Bracing could be effective for long term in selected patients with AIS; since compliance was not objectively measured, we don't feel confident to give any indication about everyday dosage.	
**Piazza 1990<br>United States**	1. Number with AIS: 76<br>2. Age at brace start:<br>mean: 12.4; range: 9.4-14.9<br>3. Cobb angle at brace start:<br>range: 20° - 39°<br>20° - 29°: n=42; 30° - 39°: n=34<br>4. Risser s: 0 - 1	Risser 4	wean: a gradual weaning to 12 h/day out of the brace continued for the following year. A regimen of usage for 8 h/day or night was then instituted for an additional year.	NA		20° - 29°(n=42):<br>improved: 10(24%);<br>without chang: 20(48%);<br>worse: 12(29%);<br>fused: 2; >40°: 3<br>30° - 39°(n=34):<br>improved: 9(27%);<br>without chang: 15(44%);<br>worse: 10(29%);<br>fused: 7; >40°: 3	The Wilmington brace appears to be an acceptable alternative to the Milwaukee brace for adolescent patients with idiopathic scoliosis. Although all patients should be observed after termination of active brace treatment, patients with double-structural curves that progressed during the bracing program may be at increased risk of continued progression	44% of the patients incurred most of their progression during the first 2 years after the bracing program.
**Rigo 2003<br>Spain**	1. Number with AIS: 1<br>2. Age at brace start: 12 years, 10 months<br>3. Cobb angle at brace start:<br>left convex thoracolumbar curve: 53°<br>a secondary right thoracic curvature: 34°<br>4. Risser s: 2	Risser 4	wean: reduce the treatment time to 16 hours first and to 8 hours afterwards. Six months later she had a stable scoliosis of thoracolumbar and thoracic, so the patient leave the brace.	thoracolumbar: 34° Cobb<br>thoracic: 31° Cobb		thoracolumbar: 36° Cobb<br>thoracic: 31° Cobb	This case report shows that, contrary to the paradigm in the international literature, an improvement of both cosmesis and curvature is possible in immature patients with AIS when conservative treatment of a certain standard is applied. The results of such treatment are appreciated by the patients because of the significant reduction of the truncal deformity as demonstrated by surface topography.	
**Upadhyay 1995<br>China**	1. Number with AIS: 85<br>2. Age at brace start:<br>The patients were graded according to outcome into two groups.<br>good (n=52): had a final Cobb angle of 45° or less and within 5° of prebrace measurement.<br>failure (n=33): Cobb angle increased 6° or more from their prebrace value at final follow-up or before surgery.<br>good: mean: 13.8 ±1.3<br>failure: mean: 12.2 ±1.3<br>3. Cobb angle at brace start:<br>range: 20° - 45°<br>good: mean: 33.0 ±7.0<br>failure: mean: 33.0 ± 5.6<br>4. Risser s: < 3	Risser 4+	wean: the weaning program included reducing the bracing time to 18 hours for the first month, then 14-16 hours/day for the next 2 months. This was followed by nightly bracing for another 2-3 months.	at post-brace:<br>good: mean: 23.0 ± 10.2<br>failure: mean: 33.0 ± 8.4		at final:<br>good: mean: 28.0 ± 8.2<br>failure: mean: 50.0 ± 8.6	The findings show a strong association between changes in vertebral rotation and the Cobb angle after application of a brace and the final outcome. Reduction in both is indicative of a good outcome, whereas increase in one or both indicates brace failure.	
**Wiley 2000<br>Lebanon**	1. Number with AIS: 50<br>2. Age at brace start:<br>mean: 13 ± 1; rang: 10 ± 1 to 16 ± 4<br>group 1: wore the brace 18–23 hours per day (full-time)<br>group 2: wore the brace more than 12 hours per day but less than 18 hours per day (part-time)<br>group 3: wore the brace less than 12 hours per day (noncompliant)<br>group 1: 12.8; group 2: 13.6; group 3: 13.1<br>3. Cobb angle at brace start:<br>mean: 38.55°; range: 35° - 45°<br>group 1: 38.0; group 2: 38.7; group 3: 39.4<br>4. Risser s: 0 - 2	At skeletal maturity (i.e., Risser sign 4 for girls and 5 for boys) and the absence of documented height gain for more than 6 months.	wean: 6 to 12 month weaning period. Usually consisting of nighttime wear only, after which bracing was discontinued.	an initial mean in-brace curve:<br>mean: 21.7°<br>rang: 0-37°<br>group 1: 19.5°; range: 0–31°<br>group 2: 21.5°; range: 6–32°<br>group 3: 26.4°; range: 19–35°	mean time out of brace was 3.1 years (range: 2–6 years):<br>mean: 41.7°; range: 14–70°<br>group 1: 35.3°; range: 14–54°<br>group 2: 42.2°; range: 27–54°<br>group 3: 53.3°; range: 32–70°	At long-term follow-up:<br>mean: 35.8°; range: 11–53°<br>group 1: 33.8°; range: 11–43°<br>group 2: 32.9°; range: 21–43°<br>group 3: 47.8°; range: 36–56°	These long-term data confirm that the Boston brace when used 18 or more hours per day is effective in preventing progression of large curves at a mean of 9.8 years after bracing is discontinued.	

**Wong 2022 Denmark**	1. Number with AIS: 15<br>2. Age at brace start:<br>mean: 12.26 (SD: 2.76)<br>3. Cobb angle at brace start:<br>mean: 16.8° (SD: 2.8) rang: 15-25<br>4. Risser s: 0 - 2	At least Risser 4, duration of menstruation of 2 years for girls, and a skeletal hand age of 14 years and 16 years for girls and boys (Sanders stage 8), respectively.	stop	mean: 11°(SD: 7.4°)		mean: 12.0° (SD: 6.8°)	The Spinaposture brace© had an immediate in-brace deformity correction and a thoracic kyphotic effect. At skeletal maturity, the deformities improved more than expected when compared to that of the natural history/observation and similar to that of other soft braces. No long-term deformity progression was seen. To substantiate these findings, stronger designed studies with additional subjects are needed.	
**Xu 2017 China**	1. Number with AIS: 488<br>2. Age at brace start:<br>mean: 13.2± 1.5; rang: 10-15<br>3. Cobb angle at brace start:<br>mean: 29.5 ± 6.6°;rang: 25-35°<br>4. Risser s: 1.5 ± 1.4; range: 0–2	At skeletal maturity: Risser sign grade 4 for girls and 5 for boys, at least 30 months since menarche for girls and absence of height increase for >6 months.	wean: No specific method of weaning is stated	NA		mean: 28.1 ± 9.2°<br>range: 21–43°	Patients with low ICR, low Risser sign, and young age could have a remarkably high risk of bracing failure. A rate of 10% was the optimal cut-off point for ICR, which can be used to effectively stratify braced patients.	failure group:<br>initial age: 12.8 ± 1.6; initial Risser sign: 1.4 ± 1.5;<br>ICR: 4.0%± 8.4%<br>success group:<br>initial age: 13.4 ± 1.6; initial Risser sign: 2.1 ± 1.5;<br>ICR: 16.3% ± 15.5%
**Yrjönen 2006 Finland**	1. Number with AIS: 72<br>2. Age at brace start: mean: 13.3 years<br>Providence night brace group: mean: 13.5 years; range: 9.3–15.2<br>Boston full-time brace group: mean: 13.0 years; rang: 10.8–15.3<br>3. Cobb angle at brace start: mean: 28.4°<br>Providence night brace group: mean: 28.4°; rang: 20–42<br>Boston full-time brace group: mean: 28.8°; rang: 20–42<br>4. Risser s: 0 - 3<br>Providence night brace group:<br>Risser 0–1: 17/36 (47%); Risser 2–3: 19/36 (53%)<br>Boston full-time brace group:<br>Risser 0–1: 18/36 (50%); Risser 2–3: 18/36 (50%)	At growth cessation and a Risser sign of at least 4	stop	NA		At the latest follow-up:<br>Providence night brace group: mean: 29.1°<br>Boston full-time brace group: mean: 28.2°	With the Providence night brace, very good in-brace correction (mean 92%) on supine radiographs was achieved. A total of 27% of the curves progressed >5 during follow-up but these results were comparable with the results of full-time Boston bracing. The results of this study suggest that the Providence night brace may be recommended in the treatment of AIS with curves <35 in lumbar and thoracolumbar cases.	An increase of Cobb angle >5°of themajor curve:<br>Providence night brace group: 10/36 (27%)<br>Boston full-time brace group: 8/36 (22%)<br>The average correction for the braces as seen on the prone radiographs was 92% for the Providence nighttime brace and in standing radiographs 50% for the Boston group. Corrections >100% were achieved in 31% of the Providence brace patients.
**Yrjönen 2007 Finland**	1. Number with AIS: 102<br>2. Age at brace start:<br>male: mean: 14.8 years; female: mean: 13.1 years<br>3. Cobb angle at brace start:<br>male: mean: 33.1°; female: mean: 32.4°<br>4. Risser s: 0 - 3	When the growth had ceased and the iliac apophyses were ossified to Risser 4.	wean: No specific method of weaning is stated	At the weaning of the brace<br>male: mean: 31.4°; female: mean: 27.0°		At the latest follow-up:<br>male: mean: 34.8°;<br>female: mean: 32.0°	The overall results of brace treatment of idiopathic scoliosis in male patients were inferior compared with matched females. One reason for inferior overall results in boys was poor compliance with brace wear. Progression of curves > 5 occurred in 31.4% of male patients, which, however, corresponds with many published results of female patients. The results of this study suggest that bracing of boys may be recommended with the same principles as girls in adolescent idiopathic scoliosis.	In the analysis of the data of the compliant boys (n=33), the age of the patient, the curve pattern or the curve magnitude did not have statistical influence on the risk of progression (P > 0.05). The importance of Risser sign remained obscure but the association between the risk of progression > 5 and correction% in brace was statistically significant (P < 0.05).
